# Effects of Hippocampal Sparing Radiotherapy on Brain Microstructure—A Diffusion Tensor Imaging Analysis

**DOI:** 10.3390/brainsci12070879

**Published:** 2022-07-04

**Authors:** Johannes G. Dinkel, Godehard Lahmer, Angelika Mennecke, Stefan W. Hock, Tanja Richter-Schmidinger, Rainer Fietkau, Luitpold Distel, Florian Putz, Arnd Dörfler, Manuel A. Schmidt

**Affiliations:** 1 Neuroradiologisches Institut des Universitätsklinikums Erlangen, Friedrich-Alexander-Universität Erlangen-Nürnberg (FAU), 91054 Erlangen, Germany; johannes.dinkel@ukr.de (J.G.D.); angelika.mennecke@uk-erlangen.de (A.M.); stefan.hock@uk-erlangen.de (S.W.H.); arnd.doerfler@uk-erlangen.de (A.D.); 2 Strahlenklinik des Universitätsklinikums Erlangen, Friedrich-Alexander-Universität Erlangen-Nürnberg (FAU), 91054 Erlangen, Germany; godehard.lahmer@uk-erlangen.de (G.L.); rainer.fietkau@uk-erlangen.de (R.F.); luitpold.distel@uk-erlangen.de (L.D.); florian.putz@uk-erlangen.de (F.P.); 3 Psychiatrische und Psychotherapeutische Klinik des Universitätsklinikums Erlangen, Friedrich-Alexander-Universität Erlangen-Nürnberg (FAU), 91054 Erlangen, Germany; tanja.richter-schmidinger@uk-erlangen.de

**Keywords:** hippocampus, diffusion tensor imaging, fractional anisotropy, cranial radiotherapy, hippocampal sparing radiotherapy, cognitive impairment

## Abstract

Hippocampal-sparing radiotherapy (HSR) is a promising approach to alleviate cognitive side effects following cranial radiotherapy. Microstructural brain changes after irradiation have been demonstrated using Diffusion Tensor Imaging (DTI). However, evidence is conflicting for certain parameters and anatomic structures. This study examines the effects of radiation on white matter and hippocampal microstructure using DTI and evaluates whether these may be mitigated using HSR. A total of 35 tumor patients undergoing a prospective randomized controlled trial receiving either conventional or HSR underwent DTI before as well as 6, 12, 18, 24, and 30 (±3) months after radiotherapy. Fractional Anisotropy (FA), Mean Diffusivity (MD), Axial Diffusivity (AD), and Radial Diffusivity (RD) were measured in the hippocampus (CA), temporal, and frontal lobe white matter (TL, FL), and corpus callosum (CC). Longitudinal analysis was performed using linear mixed models. Analysis of the entire patient collective demonstrated an overall FA_CC_ decrease and RD_CC_ increase compared to baseline in all follow-ups; AD_CC_ decreased after 6 months, and MD_CC_ increased after 12 months (*p* ≤ 0.001, 0.001, 0.007, 0.018). AD_TL_ decreased after 24 and 30 months (*p* ≤ 0.004, 0.009). Hippocampal FA increased after 6 and 12 months, driven by a distinct increase in AD_CA_ and MD_CA_, with RD_CA_ not increasing until 30 months after radiotherapy (*p* ≤ 0.011, 0.039, 0.005, 0.040, 0.019). Mean radiation dose correlated positively with hippocampal FA (*p* < 0.001). These findings may indicate complex pathophysiological changes in cerebral microstructures after radiation, insufficiently explained by conventional DTI models. Hippocampal microstructure differed between patients undergoing HSR and conventional cranial radiotherapy after 6 months with a higher AD_CA_ in the HSR subgroup (*p* ≤ 0.034).

## 1. Introduction

Although a crucial pillar of modern tumor therapy, improving morbidity and mortality, cranial radiotherapy (CR) can be associated with severe adverse reactions. While symptoms of acute (during CR) and early delayed (weeks to a few months after CR) radiation reactions are usually mild, self-limiting, and/or responsive to corticoid treatment, late-delayed reactions (several months to years after CR) can be progressive and irreversible [1]. Along with vascular complications, neuroendocrine dysfunction, and radionecrosis, neurocognitive impairment may be observed in this phase [1,2]. Symptoms range from impairment of short and long-term memory, hippocampal spatial learning, verbal learning, and intellectual decline to debilitating dementia with manifestation usually 4–6 months after radiation [3,4,5,6].

Improvements in survival due to modern therapeutic approaches give relevance to such reactions even in the context of highly malignant entities. While whole-brain radiotherapy distinctly improves cognitive symptoms and survival of patients suffering from multiple brain metastases [7], its use in the case of few cerebral metastases is increasingly controversial. Despite a profound benefit in local and distant metastasis control, as demonstrated by multiple phase III trials, the associated risk of neurotoxicity may still prove decisive for the progression of cognitive impairment [8,9,10]. While studies using the fairly insensitive Mini Mental Status Examination had reported improvements or prolonged preservation of neurocognitive abilities [11,12], more extended neurocognitive testing in further trials subsequently demonstrated severe cognitive decline [5,10]. Means of limiting cognitive impairment could therefore prove decisive when determining future treatment regimens for such patients. Besides neuroprotective adjuvant medications (e.g., memantine), proton beam radiotherapy, and stem cell therapies, the selective sparing of the hippocampi has been proposed to mitigate cognitive symptoms of CR [13,14,15].

Its crucial role in short- and long-term memory predisposes the hippocampus as a target for therapeutic sparing when aiming to limit negative cognitive side effects, particularly memory decline [16]. It is one of only two brain regions accommodating neurogenic stem cells, thus enabling adult neurogenesis, a process crucial to preserving memory capacity [17]. Depletion of the distinctly radiosensitive hippocampal stem cell population is one of the defining pathomechanisms of the cerebral radiation response [18]. Several studies suggest a causative link between impaired neurogenesis and neurocognitive decline, presumably due to chronic neuroinflammatory microglial activation and chemokine production [18,19,20]. Clinical investigations have shown dose-dependent correlations between hippocampal irradiation and neurocognitive deficits, particularly regarding verbal memory [21,22,23,24,25,26]. Furthermore, the hippocampal dose is more strongly associated with cognitive decline than the total irradiation dose [21,27]. Even after the low radiation doses used in juvenile hemangioma treatment, subtle cognitive deficits have been demonstrated to correlate with hippocampal radiation dose [28]. The risk of tumor recurrence due to hippocampal sparing is limited, given the rarity of metastases in the hippocampal region [29]. Hippocampal-sparing radiotherapy (HSR) is associated with less memory decline in comparison to historical risk estimations and, recently, in comparison to control groups undergoing Conventional Cranial Radiotherapy (CCR) [30,31,32,33].

Conventional imaging findings after CR are unspecific, rare in early phases, and can remain absent even during clinically manifest cognitive decline [34]. Using Diffusion-Tensor Imaging (DTI), subtle microstructural changes to the brain parenchyma can be detected even after low radiation doses [6]. While DTI changes of cerebral white matter following CR are well documented, pre-existent data regarding the hippocampus is sparse and sometimes conflicting. Limitations of previous research, like very limited sample sizes and incomplete DTI parameter measurements beyond FA, must be overcome to understand hippocampal microstructure changes after radiation. In this study, we aim to analyze the effects of CR on brain microstructure using DTI, particularly in the hippocampus, and to evaluate whether the microstructure of the hippocampus differs between patients after HSR and those after CCR.

## 2. Materials and Methods

### 2.1. Study Design

The study was approved by the institutional review board. The underlying trial (HIPPO-SPARE 01) was carried out in accordance with The Code of Ethics of the World Medical Association (Declaration of Helsinki) and adhered to national Good Clinical Practice guidelines. It was registered by the University of Erlangen-Nürnberg Medical School as a clinical trial (Identifier NCT01849484) on 8 May 2013 and is ongoing at the time of publication of this article. Study participants provided their written informed consent. Diagnosis, recruitment, and radiation planning were conducted by the Department of Radiation Oncology at our clinic. Briefly, HIPPO-SPARE 01 is a prospective randomized controlled trial (RCT) in which study participants were randomly assigned to two groups, one receiving CCR, and the other HSR. Inclusion criteria were a minimum age of 18 years, diseases indicating neurocranial radiotherapy confirmed by histology or imaging ([skull base] meningioma, pituitary adenoma, craniopharyngioma, or brain metastases), and Karnofsky-State ≥ 50%. Exclusion criteria included, among others, persistent drug and/or alcohol abuse, prior neurocranial radiotherapy, more than 3 brain metastases, and gross tumor volume in the hippocampal region or hippocampal avoidance zone.

The aim of this sub-investigation was to evaluate the potential of DTI to detect microstructural changes in the brain following CR and to evaluate whether these effects can be mitigated using HSR.

### 2.2. Follow-Up and Imaging Protocol

**Figure 1:**
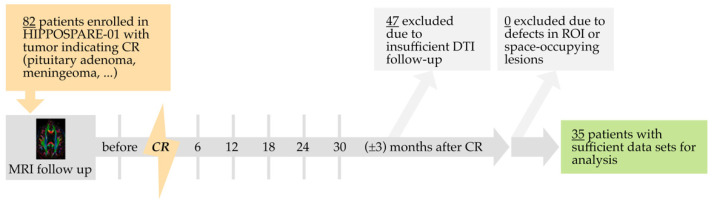
Schematic representation of study protocol illustrating MRI follow-up and patient exclusion. CR = cranial radiotherapy. ROI = regions of interest.

Follow-up intervals of 6 months (±3 months) after completion of CR were chosen (Figure 1). At each interval, a standardized MRI protocol, including DTI, was conducted. Primary outcome measures were the following DTI Parameters: Fractional Anisotropy (FA), Mean Diffusivity (MD), Axial Diffusivity (AD), and Radial Diffusivity (RD).

All MRI exams were performed using a 1.5 T Siemens Magnetom Aera Scanner with a dedicated 20-channel head/neck coil. High-resolution MRI scans of the brain were performed with a gradient field strength up to 45 mT/m (at 200 T/m/s). DTI was performed in the axial plane with 2 mm isotropic resolution using a single-shot, spin-echo, echo-planar imaging (EPI) diffusion tensor sequence (TR = 10500 ms, TE = 93 ms, readout bandwidth = 1628 Hz/pixel, FoV 256 × 256 mm^2^, acquisition matrix size 128 × 128, and spectral fat saturation). Diffusion weighting was carried out with a maximal b-factor of 1000 s/mm^2^ along 20 icosahedral directions complemented by one scan with b = 0. All DTI measurements were repeated with reversed-phase encoding to control for echo-planar imaging distortions during the postprocessing.

MRI protocol also included the acquisition of a 3-dimensional (3D) T1-MPRAGE sequence with 1 mm isotropic resolution (TR = 1900 ms, TE 3.0 ms, FoV = 250, acquisition matrix size = 256 × 256).

### 2.3. Processing and Measurement

**Figure 2:**
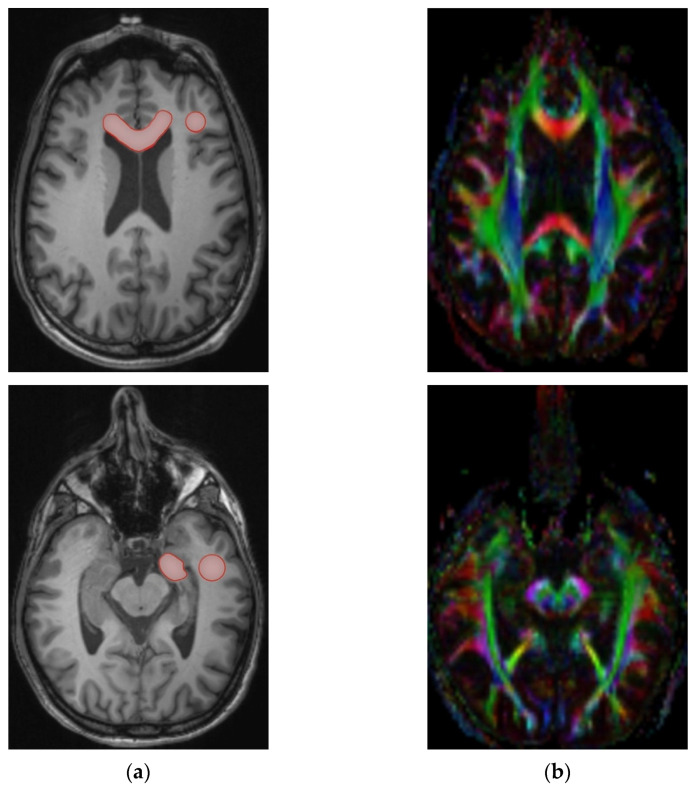
Co-registration of T1 MPRAGE data sets (**a**) and Diffusion Tensor Imaging (DTI) maps (**b**) for the anatomic region of interest (ROI) sampling. ROI marked in red: Corpus callosum, Frontal Lobe, Hippocampus, and Temporal Lobe.

Diffusion data was collected in the original and reversed phase-encoding direction, resulting in an image pair with distortions in opposite directions. DICOM images were converted to NIfTI Files (Neuroimaging Informatics Technology Initiative) using dcm2nii from the MRIcron package [35]. The susceptibility-induced off-resonance field of the image pair was estimated and applied to the co-registered image stack using a method similar to that described by Andersson et al. using the FSL TOPUP tool [36,37]. After correction for eddy-current artifacts, image data was reconverted to DICOM for further processing. The Software Olea Sphere fit a diffusion tensor model for each voxel. The scalar indices FA, MD, AD, and RD, were calculated. Anatomical regions of interest (ROI) were identified by co-registration of DTI and T1-MPRAGE data, as well as manual correction for possible residual motion artifacts to produce an optimum fit to the target area and DTI data sets. Due to its narrowing shape and proximity to the lateral ventricle, hippocampal DTI measurements are highly susceptible to cerebrospinal fluid (CSF) contamination, a momentous partial volume effect occurring in anatomic areas close to CSF [38]. To minimize the influence of this effect, hippocampal ROIs were placed only in their rostrocaudal proportion, where the diameter is largest in axial imaging (Figure 2). A visual examination was conducted for surgical or pathological defects in the regions of interest, large space-occupying lesions interfering with the ROIs, and poor image quality. Mean values of the scalar indices within the ROIs were taken bilaterally in the hippocampus (CA), temporal and frontal lobe white matter (TL, FL), and genu corporis callosi (CC).

### 2.4. Statistics

Statistical analysis was conducted using IBM SPSS (Version 26, SPSS Inc., Chicago, IL, USA). The significance level was set at *p* ≤ 0.05 (two-sided). Data analyses were of an exploratory manner. Thus, no adjustments for multiple testing were implemented. All measurements between 08/2012 and 02/2017 were included. Thus, no sample size calculation was conducted.

#### 2.4.1. Analysis of Patient Characteristics

Descriptive analyses were conducted in regard to age (years), gender, tumor entity, total radiation dose (Gy), hippocampal dose (Gy), number of fractions, dose per fraction (Gy), duration of radiotherapy (days), and history of chemotherapy, surgery, or biopsy. An Independent *t*-test was used to test the equality of the aforementioned parameters for both groups.

#### 2.4.2. Analysis of DTI Changes after Cranial Radiotherapy

Mixed linear models (MLMs; maximum likelihood method, autoregressive covariance type) were applied to evaluate all measurements of FA, MD, AD, and RD before CR, as well as in intervals up to 6, 12, 18, 24, and 30 months after CR (±3 months). MLMs use the full data set, replacing missing values by using maximum likelihood estimates. Thus, the data sets of all patients who had received MRI follow-up could be included in the analysis (*n* = 35).

#### 2.4.3. Analysis of the Effects of Mean Hippocampal Dose on DTI Changes

An analogous mixed linear model approach with the additional inclusion of a hippocampal dose as a covariate was used to analyze its effects on DTI signal changes.

#### 2.4.4. Analysis of the Effects of Hippocampal Sparing on DTI Changes

A further analogous mixed linear model was used to compare the longitudinal DTI measurements of the individual subgroups undergoing CR with and without hippocampal sparing (main effects and interaction effects between time after CCR/HSR).

## 3. Results

### 3.1. Patient Characteristics

A total of 82 subjects undergoing cranial radiation were registered for the HIPPO-SPARE 01 trial at the time of analysis. Of these, 47 patients could not be included in the analysis as radiotherapy was incomplete or they had not yet received DTI after radiotherapy. The studies of the remaining 35 patients were examined visually for surgical or pathological defects in the regions of interest, large space-occupying lesions interfering with ROI, and poor image quality, in the process of which no further studies had to be excluded. Infrequent deviations in patient attendance to follow-up MRI reduced the number of data sets available for analysis at the individual time points, as detailed in Table 1.

Table 1 describes the patient characteristics of the 35 individuals that met the study criteria. There was a female predilection (20 vs. 15). The majority of tumor entities indicating radiotherapy were meningiomas or pituitary adenomas. Cranial radiation was performed over an average of 41 (±6) days with a mean total dose of 49.3 (±3.4) Gy. The bilateral mean total hippocampal dose was 10.9 Gy (±9.0) and was significantly lower in the HSR subgroup compared to CCR (6.9 ± 4.3 vs. 16.2 ± 10.9; *p* ≤ 0.006). Both groups did not differ significantly in size, age, gender, history of chemotherapy/surgery/biopsy, or baseline DTI parameters (FA, MD, AD, and RD).

### 3.2. DTI Changes after Cranial Radiotherapy

**Figure 3:**
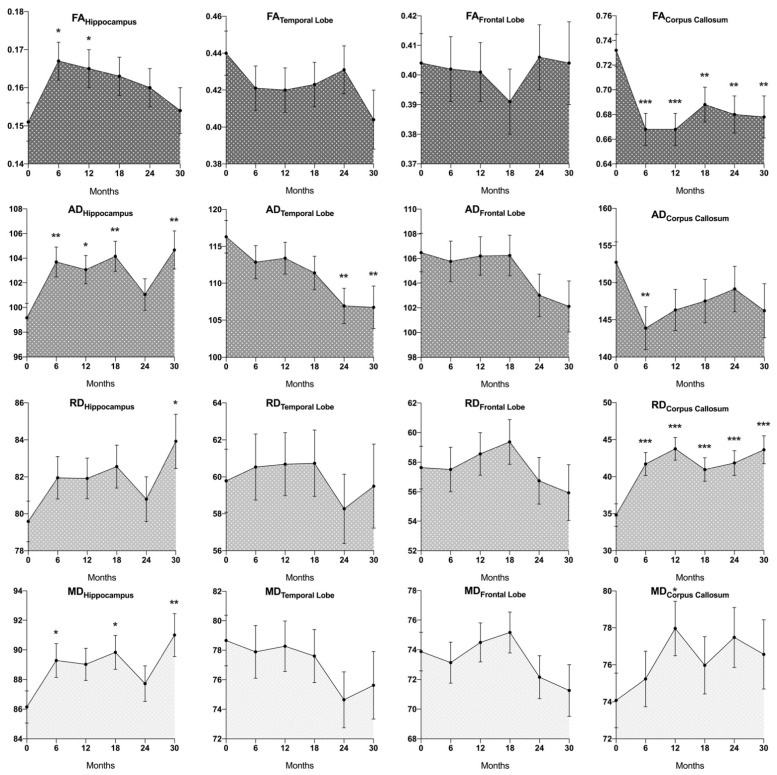
Mean values of fractional anisotropy (FA), axial diffusivity (AD), radial diffusivity (RD), and mean diffusivity (MD) before cranial radiotherapy (CR) as well at prospective intervals of 6 (± 3) months after CR. Baseline measurements (before CR) are represented as “0” on the x-axis. In the corpus callosum, with its longitudinally structured white matter tracts, FA decreased after CR, driven by an increase in RD and a decrease in AD. This effect appears to attenuate over time. Similar, yet only partially significant, tendencies were observed in temporal and frontal lobe white matter. In the hippocampus, however, driven by an unexpected increase in AD, a distinct increase in FA was observed, followed by a return to near-normal levels after 30 months. Differences compared to baseline: * = *p* ≤ 0.05, ** = *p* ≤ 0.01, *** = *p* ≤ 0.001. Error bars = ±1 standard deviation.

DTI signal changes in the hippocampus, temporal lobes, frontal lobes, and corpus callosum of all patients over time are depicted in Figure 3.

In cerebral white matter, an overall decrease of FA_CC_ (*p* < 0.001) and increase of RD_CC_ (*p* ≤ 0.001) was observed when comparing measurements before and after CR, which was significant in all individual follow-up measurements when compared to baseline (FA after 6, 12, 18, 24, and 30 months (±3): *p* < 0.001, *p* < 0.001, *p* ≤ 0.009, *p* ≤ 0.004, *p* ≤ 0.009; respective RD: *p* < 0.001, *p* < 0.001, *p* ≤ 0.001, *p* < 0.001, *p* < 0.001). AD_CC_ tended to decrease after CR, which was significant after 6 months (*p* ≤ 0.007). MD_CC_ tended to increase after CR, which was significant 12 months after radiotherapy compared to baseline (*p* ≤ 0.018) as well as when comparing measurements 12 months and 6 months after CR (*p* ≤ 0.044). In the temporal lobe white matter, AD decreased overall after CR, which was significant 24 and 30 months after CR compared with baseline (*p* ≤ 0.004, *p* ≤ 0.009), as well as when comparing 24 months to 12 months post CR (*p* ≤ 0.032). MD_TL_ and FA_TL_ showed a decreasing tendency over time, which did not reach significance (e.g., FA_TL_ after 30 months compared to baseline: *p* ≤ 0.078; MD_TL_ after 24 months: *p* ≤ 0.116). Similarly, AD showed a decreasing tendency in the frontal lobe, which did not reach significance (e.g., AD_FL_ after 30 months: *p* ≤ 0.094). No distinct changes were observed in RD_TL_, AD_TL_, FA_FL_, MD_FL_, or RD_FL_.

Hippocampal FA had considerably increased 6 months (*p* ≤ 0.011) as well as 12 months (*p* ≤ 0.039) after CR when compared to baseline. After the peak increase at 6 months, FA showed a gradual tendency to decrease, with differences to baseline becoming insignificant after 18 months (18, 23, and 30 months compared to baseline: *p* ≤ 0.086, *p* ≤ 0.222, *p* ≤ 0.734). AD_CA_ and MD_CA_ were overall considerably higher after CR (*p* ≤ 0.005, *p* ≤ 0.040), with significantly higher AD_CA_ at the individual time points 6, 12, 18, and 30 months after radiation compared to baseline (*p* ≤ 0.003, 0.015, 0.003 and 0.005) and higher MD_CA_ 6, 18 and 30 months after CR (*p* ≤ 0.026, 0.020 and 0.008). There was an increasing tendency of RD_CA_ after CR, which was significant 30 months after radiation (*p* ≤ 0.019).

### 3.3. Effects of Mean Hippocampal Dose on DTI Changes

There was a pronounced positive correlation between mean hippocampal radiation dose and FA_CA_ after radiotherapy (*p* < 0.001)—FA_CA_ values were 0.001024 higher per Gy of radiation (0.68% of mean baseline FA_CA_ per each Gy of radiation dose). No significant interactions between mean hippocampal dose and hippocampal AD, MD, or RD were found.

### 3.4. Effects of Hippocampal Sparing on DTI Changes

**Figure 4:**
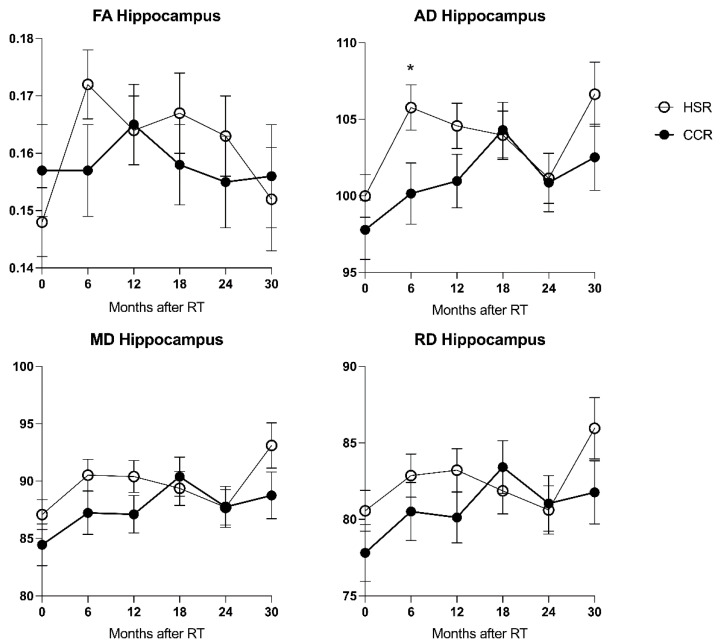
Hippocampal Sparing Radiotherapy (HSR) vs. Conventional Cranial Radiotherapy (CCR). Higher hippocampal AD was noted in the HSR subgroup and 6 months after CR. MD was higher in the HSR subgroup with a tendency to significance (*p* ≤ 0.072). Further differences were insignificant. Differences compared to baseline: * = *p* ≤ 0.05. Error bars = ±1 standard deviation.

Before radiation, there were no significant differences in hippocampal FA, MD, AD, or RD of subjects in the CCR and HSR subgroups. After CR, AD_CA_ of the HSR subgroup was higher overall (*p* ≤ 0.034) as well as in the individual follow-up measurements 6 months after radiation (*p* ≤ 0.025) when compared to the CCR subgroup (Figure 4). MD_CA_ was higher overall in the HSR subgroup with a tendency to significance (*p* ≤ 0.072). There were no significant differences between FA_CA_ and RD_CA_ of both groups.

## 4. Discussion

To the knowledge of the authors, this is the first study based on a prospective randomized controlled trial (1) analyzing multiple DTI measurements of the hippocampus over an extended period in a comparatively large data set and (2) evaluating HSR in direct comparison to conventional CR using this technique.

Our patient cohort consisted predominantly of meningioma and pituitary adenoma patients. Cognitive impairment has been described even after the moderate radiation doses used in radiotherapy of benign CNS tumors, tumors of the nasopharynx, or in prophylactic indications [21,39,40,41]. Benign entities, however, are associated with fewer confounders than malignancies (i.e., growth dynamic, edema, mass effect, medication). This implies particular suitability of the studied cohort for evaluating the cerebral radiation response pathophysiology, as well as improved comparability to preclinical data, mostly derived from healthy individuals [42].

This study focuses on the late-delayed phase of radiation reactions (more than 12 weeks after CR), during which cognitive decline is usually observed. To limit the confounding influence of the distinctly different, likely edema-driven, pathophysiological processes underlying acute and early delayed injury, follow-up intervals of 6 months (±3 months) after CR were chosen, thus excluding measurements in the first 3 months after CR.

Typical cerebral white matter changes after CR include a decrease in FA [6,43,44,45,46,47,48], as well as increases in MD [6,43,44,46,48] and RD [6,43,44,45,46,47,48]. Regarding AD, both decreases and increases have been reported [6,43,44,46]. Pediatric studies have reported lower FA and higher MD in the white matter of long-term radiotherapy survivors compared to healthy controls [49,50].

We detected similar DTI changes in cerebral white matter after CR, especially in the corpus callosum, with a FA decrease and RD increase at all time intervals after CR, an MD increase after 12 months and AD decrease after 6 months, as well as in the temporal lobe with a decrease in AD after 24 and 30 months. The regional variability of DTI changes following CR detected in our patient cohort bears further similarities to previous reports, where most prominent changes have been reported (along with cingulum and fornix) in the corpus callosum, while various other white matter regions had not shown significant alterations (or even opposite parameter movements) [46]. Previous studies proposed topographical variability in radiosensitivity to explain this phenomenon, with a possible predilection for late myelinating neural cells [46,51]. It stands to reason that the pronounced DTI changes measured in certain white matter regions, particularly the corpus callosum, result at least in part from the intrinsically high absolute DTI signal of these regions (Figure 3), likely due to their large proportion of closely and parallelly aligned white matter tracts.

Sparse data are available regarding the radiation response of grey matter and the hippocampus, with conflicting results in preclinical and clinical trials. Clinical studies reported either decreasing or insignificant changes in FA and MD [50,52,53], restricted, however, by small case numbers and lacking measurement of further diffusion parameters. In an investigation of 25 primary brain tumor patients, an increase in hippocampal FA was associated with verbal and memory decline and an increase in hippocampal MD with verbal decline [54]. Preclinical studies of acute to early delayed radiation response reported either constant and decreasing FA values [55,56,57,58], either decreasing or increasing AD and MD [55,56,58], and an increase of RD [55]. One study showed significantly higher FA values in rats 1-9 months after irradiation with 39 Gy compared to controls [59].

In our patient cohort, a significant increase in hippocampal FA was noted 6 and 12 months after CR, followed by a tendency to return to near baseline levels, with differences to baseline becoming insignificant 18 months after CR. The FA increase was dose-dependent (0.68% increase of FA per Gy of radiation). It was driven by a strong and early increase of AD (significant at months 6, 12, 18, and 30 after CR) contrasted by a relatively late and less pronounced increase in RD (significant only 30 months after CR). While DTI changes in white matter may be explained to a large extent by axonal damage and demyelination, the demonstrated hippocampal DTI changes may indicate further, hitherto insufficiently understood, pathomechanisms of radiogenic microstructural change influencing cerebral DTI signal.

Alongside direct DNA damage and apoptosis of glial/neuronal cells, the cerebral radiation reaction consists of a complex interplay of microglial activation, astrocyte proliferation, oligodendrocyte loss and demyelination, stem cell depletion, neuronal receptor alterations, and vascular damage [3,60]. These pathophysiological phenomena are assumed to be linked by the process of chronic neuroinflammation [4,42,60]. Radiogenic cell damage leads to the activation of microglia and astrocytes [61]. Pro-inflammatory cytokines, chemokines, and reactive oxygen/nitrogen species maintain inflammation yet may lead to further oxidative stress and cell damage [62]. Furthermore, cytokines increase the penetrability of the blood-brain and the blood-liquor barrier for immigrating immune cells, leading to an unusually high number of antigen-presenting cells in the brain parenchyma [4]. They also activate local dendritic cells, which migrate to cervical lymph nodes to activate T-cells. Insufficient inhibition of this state of inflammation, i.e., due to inadequate penetration of T-lymphocytes and macrophages limiting inflammation, can lead to chronic neuroinflammation [4]. Preclinical models have shown such persistent glial activation even months after CR [63,64].

The process of extracellular waste clearance from the brain interstitium, termed the “glymphatic system”, consists of a directional proton motion along the perivascular spaces, through the interstitium, and into paravenous drainage pathways [65]. MRI models have shown glymphatic system activity throughout the brain, including the hippocampi [66,67]. Other processes associated with chronic neuroinflammation, such as M. Alzheimer’s and ischemia, have been linked with alterations in glymphatic system activity [68,69]. The influence of this systematic fluid movement through the interstitium, constituting 12–20% of cerebral water content [70], on DTI-signal is has not been systematically evaluated. We propose the hypothesis that a proportion of the DTI signal is explained by directional interstitial proton movement due to glymphatic system activity. Alterations of this system due to chronic neuroinflammation could account for the distinct increase of hippocampal MD and AD, comparatively smaller increase in RD, and consecutive decrease in FA detected in our patients. While such changes may not be limited to the hippocampus, their effect on DTI signal would likely be more apparent in less structured tissue, i.e., the hippocampi or grey matter, in comparison to cerebral white matter with its longitudinal and parallel fiber tracts, where the traditional tubular axon model conclusively explains DTI parameter patterns during microstructural change. Targeted research is needed to further explore this concept.

The compartment of perivascular spaces (PVS) has already been shown to systematically influence DTI signals [71]. Predominant measurement of DTI signal along the PVS has been proposed as a model for detecting glymphatic activity [72]. Diurnal alterations of DTI parameters could be explained in another model by liquor-like portions of the DTI signal, presumably representing PVS [73]. While the PVS of superficial brain parenchyma near the entrance point of penetrating vessels was associated with a higher MD and lower FA [71], a continuously decreasing diameter up to capillary level in deeper laying tissue, such as the hippocampus, could result in a relatively greater increase in AD and thus FA increase.

Predominant damage to the relatively isotropic, radiosensitive neuronal stem cells, as compared to the anisotropic neuronal axons, may further contribute to a FA increase. Extensive radiogenic effects on the extracellular matrix may also lead to an increase in MD and RD, and in close proximity, the PVS, AD, and FA [74].

As described above, previous studies reported decreases in hippocampal FA in measurements conducted very early after radiation. However, acute radiation reactions are thought to be driven primarily by subtle parenchymal edema not yet detectable by conventional imaging. Edema may lead to a decrease in FA both directly [75] and indirectly by restricting fluid motion through perivascular pathways [76]. Early FA decreases would therefore be expected to diminish after edema resolution in the late-delayed reactions measured in this study.

Mean hippocampal radiation dose was associated with a stronger increase in hippocampal FA. However, hippocampal DTI changes appeared to be more pronounced in the HSR subgroup. This was significant when comparing AD of both groups after 6 months, with a higher AD detected in the HSR group. The underlying mechanism of this phenomenon is unclear. Potentially, a divergent immunocellular response above a certain threshold of cellular damage might influence hippocampal DTI characteristics. The observed differences may also result from radiogenic changes outside the hippocampus. A thus triggered generalized inflammatory response triggered could impact hippocampal DTI characteristics more strongly than local radiogenic tissue damage. Notably, in this context, hippocampal sparing radiotherapy planning may lead to differences in relevant characteristics of the three-dimensional dose distribution outside the hippocampus, including increased dose inhomogeneity and higher maximum point doses.

Additionally, tissue compression due to peritumoral mass effect, the influence of crossing fiber tracts, anisotropy increases due to gliosis, and diffusion differences in the pathophysiological stages of axonal degeneration have been discussed to account for unexpected DTI changes after radiation [46,77,78,79,80].

Notably, a multilinear model approach without multiple testing corrections was chosen in this study to allow for a more direct interpretation of the observed data, which needs to be considered when interpreting the provided results. Albeit somewhat larger than previous DTI study samples evaluating hippocampal radiation response, this study is still limited by its relatively low number of subjects. Generally, the susceptibility of hippocampal measurement to CSF contamination may variably lead to false low FA values. Although reducing standardization compared to the automated selection, a manual anatomical approach to ROI selection was thus chosen to enable visual quality control by the researcher. As a technical limitation, direct co-registration of DTI-maps and the three-dimensional radiotherapy dose distribution was impossible.

Previous trials have consistently reported correlations between DTI signal alterations of cerebral white matter and neurocognitive decline [81,82], in some instances proposing a predictive nature of early DTI measurements [81,83], as well as between hippocampal dose and neurocognitive decline [16,21,22,23,26,28]. Therefore, the dose-dependent hippocampal DTI changes observed in this study suggest a potential link between radiogenic DTI changes and neurocognitive impairment, which needs to be evaluated in further research.

Differentiating which proportion of DTI signal changes result from axonal injury or demyelination and which are accounted for by further pathophysiological processes (such as glymphatic system activity, edema, vascular injury, or other inflammatory processes) will pose a crucial goal in future DTI research aiming to evaluate cerebral radiation response.

## 5. Conclusions

This study adds to evidence of DTI parameter changes in the brain parenchyma after CR, underlining the feasibility of this technique in assessing cerebral radiation response. The discovered patterns of hippocampal microstructural change indicate hitherto insufficiently explained pathomechanisms of the radiation response influencing DTI signal. 6 months after CR, hippocampal microstructure differed between HSR and CCR patients.

## Figures and Tables

**Table 1:** Patient characteristics.

	All Patients (*n* = 35) (SD)	[%]	Patients in CCR Arm (*n* = 15)	[%]	Patients in HSR Arm (*n* = 20)	[%]
Age in years						
Median	56 (±12)		52 (±8)		59 (±14)	
Range	29–77		43–75		29–77	
Sex						
Female	20	[57]	8	[53]	12	[60]
Male	15	[43]	7	[47]	8	[40]
Tumor Entity						
Meningioma	18	[51]	10	[67]	8	[40]
Pituitary Adenoma	12	[34]	5	[33]	7	[35]
Meningioma and Pituitary Adenoma	1	[3]	0	[0]	1	[5]
Craniopharyngioma	2	[6]	0	[0]	2	[10]
Small Cell Lung Cancer	1	[3]	0	[0]	1	[5]
Large Cell Neuroendocrine Carcinoma	1	[3]	0	[0]	1	[5]
Radiotherapy						
Definitive Stereotactic	23	[66]	12	[80]	11	[55]
Postoperative Stereotactic	10	[29]	3	[20]	7	[35]
Definitive Whole Brain Radiotherapy	1	[3]	0	[0]	1	[5]
Postoperative Whole Brain Radiotherapy	1	[3]	0	[0]	1	[5]
Total Dose in Gy						
Mean (±SD)	49.3 (±3.4)		50.0 (±2.2)		48.7 (±4.0)	
Range	38.0–54.0		45.0–52.2		38.0–54.0	
Mean number of fractions	27 (±2)		28 (±1)		27 (±3)	
Mean dose per fraction (Gy)	1.8		1.8		1.8	
Duration (days)	41 (6)		42 (3)		41 (8)	
Duration Range	24–66		37–48		24–66	
Hippocampal Dose in Gy						
Mean (SD)	10.9 (9.0)		16.2 (10.9)		6.9 (4.3)	
Range	0.1–39.2		2.0–39.2		0.1–14.5	
Chemotherapy						
yes	4	[11]	1	[7]	3	[15]
none	31	[89]	14	[93]	17	[85]
Cerebral Surgery						
yes	32	[91]	14	[93]	18	[90]
none	3	[9]	1	[7]	2	[10]
Cerebral Biopsy						
yes	3	[9]	0	[0]	3	[15]
none	32	[91]	15	[100]	17	[85]
Mean Hippocampal Baseline DTI Values						
Fractional Anisotropy (±SD)	0.15 (±0.01)		0.16 (±0.01)		0.15 (±0.01)	
Mean Diffusivity (±SD)	86.1 (±1.1)		84.5 (±1.8)		90.5 (±1.4)	
Axial Diffusivity (±SD)	99.1 (±1.2)		97.8 (±1.9)		100.0 (±1.4)	
Radial Diffusivity (±SD)	79.6 (±1.1)		77.8 (±1.9)		80.6 (±1.3)	
Availability of Complete MRI Data Sets						
before CR	29		10		19	
6 ± 3 months after CR	24		8		16	
12 ± 3 months after CR	27		11		16	
18 ± 3 months after CR	24		10		14	
24 ± 3 months after CR	22		9		13	
30 ± 3 months after CR	15		7		8	

CCR = conventional cranial radiotherapy; HSR = hippocampal sparing radiotherapy.

## Data Availability

The data sets used and/or analyzed during this study are available from the corresponding author upon reasonable request.
